# Comparison of Phytoremediation Potential of *Nerium indicum* with Inorganic Modifier Calcium Carbonate and Organic Modifier Mushroom Residue to Lead–Zinc Tailings

**DOI:** 10.3390/ijerph191610353

**Published:** 2022-08-19

**Authors:** Rongkui Su, Qiqi Ou, Hanqing Wang, Yiting Luo, Xiangrong Dai, Yangyang Wang, Yonghua Chen, Lei Shi

**Affiliations:** 1 School of Environmental Science and Engineering, Central South University of Forestry and Technology, Changsha 410004, China; 2 Hunan First Normal University, Changsha 410205, China; 3 PowerChina Zhongnan Engineering Corporation Limited, Changsha 410004, China; 4 College of Geography and Environmental Science, Henan University, Kaifeng 475004, China; 5 Henan University of Engineering, Zhengzhou 451191, China

**Keywords:** lead–zinc tailings, *Nerium indicum*, calcium carbonate, mushroom residue, phytoremediation potential

## Abstract

At present, the application of phytoremediation technology in the ecological remediation of heavy metal tailings is receiving more and more attention. In this study, the physiological and biochemical response and tolerance mechanism of woody plant *Nerium indicum* to Pb and Zn under different proportions of inorganic modifier calcium carbonate (C1: 5%, C2: 10%, C3: 15%) and organic modifier mushroom residue (M1: 10%, M2: 20%, M3: 30%) was compared. The results showed that the pH value has a trend of C group > M group > CK group and organic matter has a trend of M group > CK group > C group. Phosphatase activity and catalase activity has a trend of M group > C group > CK group, but catalase was more vulnerable to the calcium carbonate concentration. Both modifiers can promote the transformation of Pb, Zn, Cu, and Cd in tailings to more stable organic bound and residual states. However, the stabilization effect of mushroom residue is better, and its stability is Pb, Zn > Cd, Cu. Both modifiers can increase the biomass of *Nerium indicum* and the modification effect of mushroom residue is better than calcium carbonate. Pb/Zn content and accumulation in *Nerium indicum* organs showed root > stem > leaf in all groups. Compared with the CK group, the enrichment coefficient of Pb/Zn in C1 and M1 groups decreased, while the translocation factor of Pb/Zn in C1 and M1 groups increased. With the increase in modifier concentration, the enrichment coefficient increases about 1.75~52.94%, but the translocation factor decreases rapidly (20.01~64.46%). Clearly, both the calcium carbonate and mushroom residue amendment could promote the growth ability of *Nerium indicum* in lead–zinc tailings and strengthen the phytoremediation potential.

## 1. Introduction

The prevention and control of heavy metal pollution is one of the research hotspots in the environmental field [1,2]. Lead–zinc tailings are solid wastes discharged from the beneficiation process after the mining of non-ferrous metal mines. After being discharged, they are generally stacked in the tailings pond, which forms a tailings wasteland after being closed [3,4]. Lead–zinc tailings have the characteristics of high metal content, poor nutrition and poor permeability. They not only occupy a lot of land, but also have multiple metal toxicity, which makes it difficult for plants to grow [5,6]. At the same time, tailings and their wastes enter the water environment and soil environment through surface runoff, leaching and seepage [7,8]. Heavy metals in the soil will destroy plant root tissue and functional characteristics, and enrich in animals and plants through the food chain [9], and then enter the human body, causing varying degrees of damage to the nervous, reproductive and digestive systems [10,11]. Heavy metal soil pollution has become an important factor endangering the safety of the ecological environment and human health in and around the mining area, and it is an environmental problem that needs to be urgently solved [12,13].

At present, the restoration of lead–zinc tailings mainly involves matrix improvement and phytoremediation. In terms of matrix improvement, some studies have shown that the type and proportion of improvers had great differences in the occurrence morphology of Pb and Zn. The 2% sepiolite significantly reduced the content of acid extractable Zn in soil but had little effect on Pb [14]. However, 2% bone char significantly reduced the content of acid extractable Pb in soil, with little influence on Zn [14]. At present, the main modifiers are calcium-rich substances [15], acid–base regulators [16], chelating agents, organic acids, and surfactants [17], sludge [18], and peat soil [19]. The mushroom residue exhibited many of the characteristics typical of other organic waste byproducts, such as high porosity and organic matter content [20]. Studies have shown that mushroom residue could improve the physical and chemical properties of tailing soil and promote the growth of ryegrass [21]. Calcium carbonate and sepiolite can reduce the content of heavy metals in different parts of rice [22]. The application of modifiers can promote the efficiency of phytoremediation in tailings.

Traditionally, hyperaccumulator plants have been used for phytoremediation. Phytoextraction is the major phytoremediation approach for removal of metals with hyperaccumulators [23]. Although hyperaccumulators have a large transfer factor, their enrichment element is relatively separate, biomass and the accumulation of heavy metals is small, and the cycle is long. These lead to the unsatisfactory effect of hyperaccumulators in repairing lead–zinc slag [24,25]. The latest research focus is woody plants [26,27,28], which are generally tolerant to heavy metals and less stressed by heavy metals, have large biomass, low content of heavy metals in their bodies, and have a large total accumulation of plants. Therefore, the method of combining woody plants with adding modifiers can be used to repair the contaminated soil. *Nerium indicum*, a tolerant woody plant, is an evergreen erect shrub with strong adaptability and strong resilience [29]. The leaves of *Nerium indicum* have a strong ability to accumulate heavy metals. In a polluted environment, the leaves of *Nerium indicum* can enrich fluorine, chlorine, sulfur, mercury, and other trace elements [30]. *Nerium indicum* is a woody plant with a fast growth rate, a large biomass and a certain economic value. Although the manganese content in the body is lower than that of the super-rich plant, the biomass is much greater than that of the super-rich plant, and the heavy metals in its body. The amount of accumulation also has an absolute advantage [31]. Therefore, the method of combining woody plant *Nerium indicum* and adding modifier can be used to repair contaminated soil.

Both organic and inorganic modifiers can improve the phytoremediation potential of heavy metal tailings. However, the mechanistic differences between these two modifiers involved in the phytoremediation of heavy metal tailings remain unclear. At present, there are relatively few studies on the restoration of lead–zinc tailings of tolerant woody plants under matrix improvement. To solve this problem, the physiological biochemical response and tolerance mechanism of woody plant *Nerium indicum* to Pb and Zn under different proportions of inorganic modifier calcium carbonate (CK: 0%, C1: 5%, C2: 10%, C3: 15%) and organic modifier mushroom residue (M1:10%, M2: 20%, M3: 30%) was compared. This study is intended to: (1) comparatively investigate the effect of inorganic modifier calcium carbonate and organic modifier mushroom residue added in lead–zinc tailings on the physical and chemical properties, including pH value, organic matter content, and soil enzyme activity; (2) explore the response of calcium carbonate and mushroom residue modifiers and their gradients on the morphology of heavy metals in lead–zinc tailings; and (3) explore the effect of calcium carbonate and mushroom residue modifiers and their gradients on growth and tolerance of *Nerium indicum*, including biological increment, heavy metal content and accumulation, bioconcentration factor, translocation factor, and transfer factor of *Nerium indicum* in lead–zinc tailings. This study attempts to provide a theoretical reference and amended materials for lead–zinc tailings restoration by woody plants.

## 2. Materials and Methods

### 2.1. Experimental Materials

The tested plant was purchased from a nursery in Hunan province as annual seedlings. Seedlings of similar height (12 cm) were selected from the same group for standby use. Lead–zinc tailings were collected from a lead–zinc tailings pond in Hunan. Calcium carbonate, mushroom residue, and calcium magnesium phosphate fertilizer were purchased from the Changsha flower market in Hunan.

### 2.2. Experimental Design

The experiment was carried out in the nursery of the biological science building of Central South University of Forestry and Technology (CSUFT). The experiment started in May 2019 in the Nursery base of CSUFT, Changsha, Hunan Province, which is located in the subtropical monsoon climate zone with obvious continental climate characteristics and an average temperature of 25 °C during the experimental period. Lead–zinc tailings were mixed with mushroom residue and calcium carbonate, respectively, in different proportions to form potted substrates in Table 1. Each basin contained 22 kg of reserves and three plants were planted in each basin. Three replicates were set in each gradient. The trial period was 1 year. Plants grew under natural conditions. The basic physical and chemical properties of the matrix are shown in Table 2.

### 2.3. Determination Method

#### 2.3.1. Physical and Chemical Properties of Tailing

The pH value of tailings was measured using the method below. Weigh 10.0 g of tailing that has passed through a 2 mm sieve into a 50 mL-tall beaker, add 25 mL of carbon dioxide-free water or calcium chloride, stir vigorously with a glass rod for 1–2 min, let it stand for 30 min, and measure with a pH meter. Organic matter content in tailings was determined by hydrated hot potassium dichromate oxidation–colorimetric method [32]. The improved BCR three-step extraction method was used to determine the content of Pb and Zn in various forms of tailings [33]. The catalase activity was determined by potassium permanganate titration, and the phosphatase activity was determined by benzyl disodium phosphate [34].

#### 2.3.2. Biomass Increment, Enrichment, and Transport of Heavy Metals of Plants

The biomass of plants is determined by subtracting the dry weight at harvest from the dry weight before planting. The content of Pb, Zn, Cu, and Cd in plants and substrates was determined by flame atomic absorption spectrophotometer. The plant samples were digested by the “nitric acid–perchloric acid” electrothermal plate digestion method. Root soil samples were digested by the “aqua regia–perchloric acid” electrothermal plate digestion method [35].

#### 2.3.3. Data Analysis

The data are all mean ± standard deviation. Excel was used for basic data processing. All results were tested by one-way ANOVA using the SPSS 19.0 (OriginLab Corporation, Northampton, MA, USA) statistical package. All figures were drawn using the Origin 2018 statistical package. The Duncan test at 5% probabilities was performed for later comparison to test for treatment differences. Different lowercase letters in the following figure and table indicate significant differences among different treatment groups. Bioconcentration factor (BCF), translocation factor (TLF), and transfer factor (TSF) of heavy metals were calculated as follows [36]:

BCF = heavy metal content in plant/heavy metal content in root soil(1)

TLF = heavy metal content of aboveground part of plants/heavy metal content of underground part of plants(2)

TSF = uptake of aboveground parts of plants/total uptake of plants (3)

## 3. Results

### 3.1. Effects of Different Modifiers and Their Gradients on the Basic Characteristics of Tailing

#### 3.1.1. Effects of Different Modifiers and Their Gradients on the pH of Tailing

**Figure 1:**
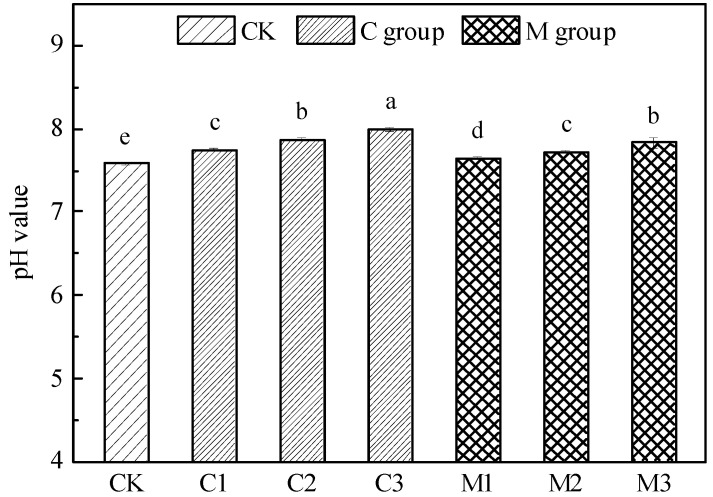
pH value in tailings treated with modifier.

As shown in Figure 1, the application of both calcium carbonate and mushroom residue can improve the pH value of tailing, and the improvement effect is proportional to the proportion of the modifier. The improvement effect of calcium carbonate is better than that of mushroom residue. After adding different modifiers, the pH value of tailings showed a trend of calcium carbonate improvement groups > mushroom residue improvement groups > the control group (*p* < 0.01), the pH values of the improvement groups were significantly higher than those of the control group. With the increase in the proportion of the modifier, there were significant differences in the increase in pH among the three improved gradients in the improvement groups. The pH value of 15% calcium carbonate treatment increased the most.

#### 3.1.2. Effects of Different Modifiers and Their Gradients on the Organic Matter of Tailing

**Figure 2:**
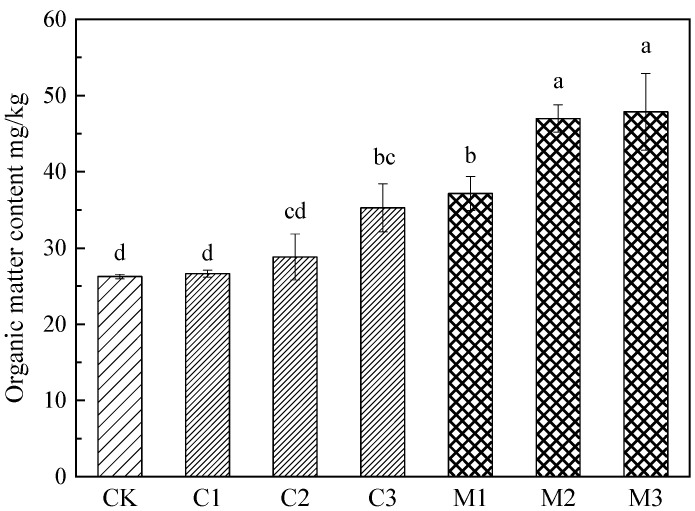
Content of organic matter in tailings treated with modifier.

The addition of two modifiers can effectively improve the organic matter content in tailing, and the effect of mushroom residue is better than that of calcium carbonate in Figure 2. Under different modifier gradients, the organic matter content in tailings showed a trend of mushroom residue improvement groups > calcium carbonate improvement groups > the control group (*p* < 0.01), and the organic matter content of mushroom residue improvement groups was significantly higher than that of the control group (1.42–1.82 times). The effect of increasing the proportion of the two modifiers on the content of organic matter in tailings is different. With the increase in proportion, there was a significant difference between 10% and 20% improvement gradients of mushroom residue improvement groups, but there was no significant difference between calcium carbonate improvement groups. The highest content of organic matter was treated with 30% mushroom residue.

#### 3.1.3. Effects of Different Modifiers and Their Gradients on Soil Enzyme Activity

**Figure 3:**
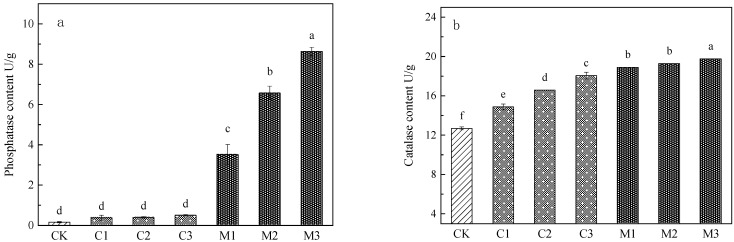
Enzyme activity in tailings: (**a**) phosphatase activity, (**b**) catalase activity.

It can be seen from Figure 3 that both modifiers can improve the activities of phosphatase and catalase in tailing, but the effect of mushroom residue is more significant. Under different improvement gradients of the two modifiers, the phosphatase activity and catalase activity in the tailings showed a trend of mushroom residue improvement groups > calcium carbonate improvement groups > the control group (*p* < 0.01) in Figure 3a,b. The activity of phosphatase and catalase in mushroom residue improvement groups was significantly increased (phosphatase, 22.00–54.00 times and catalase, 1.50–1.56 times), while only the activity of catalase was significantly increased in calcium carbonate improvement groups (catalase, 1.17–1.42 times).

In terms of the influence on phosphatase activity, with the increase in the improved proportion, there was no significant difference among the three groups of improvement gradients of the calcium carbonate group (*p* > 0.05). However, there was a significant difference among the three groups of improvement gradients of the mushroom residue group (*p* > 0.05). This indicated that the increase in calcium carbonate proportion could not significantly improve the phosphatase activity, while the increase in mushroom residue proportion could significantly improve the phosphatase activity.

In terms of the influence on catalase activity, with the increase in the improved proportion, there were significant differences among the three improvement gradients of the calcium carbonate improvement groups. This indicated that the increase in calcium carbonate proportion could significantly improve catalase activity. There was no significant difference between the treatment with 10% and 20% of mushroom residue, but there was a significant difference between the treatment with 30% of mushroom residue and the first two groups. This indicated that the increase in mushroom residue proportion had little influence on the improvement in catalase activity.

### 3.2. Effects of Different Modifiers and Their Gradients on the Morphology of Heavy Metals in Tailings

**Figure 4:**
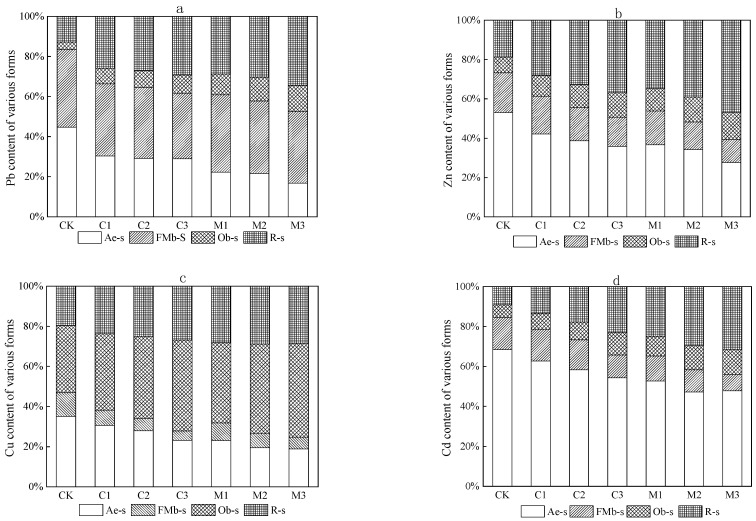
Heavy metal form content in tailings under improved treatment: (**a**) Pb, (**b**) Zn, (**c**) Cu, (**d**) Cd. acid-extractable state (Ae-s), Fe-Mn binding state, organic binding state, and residue state (R-s).

The morphological content of heavy metals in the tailings under improved treatment is shown in Figure 4. In the control group, Pb mainly exists in the acid-extractable state and Fe-Mn binding state in Figure 4a. Zn and Cd mainly exist in the acid-extractable state, and Cu mainly exists in the acid-extractable state and organic binding state in Figure 4b–d. After modified treatment of the two kinds of modifier, the content of Pb, Zn, Cu, and Cd showed a phenomenon of a significant decrease in the acid-extractable state and a significant increase in the residue state, and a small decrease in the Fe-Mn binding state and a small increase in the organic binding state. The decreased range of acid-extractable state content and the increased range of organic binding state and residue state content of four metals in mushroom residue improvement groups were higher than those in calcium carbonate improvement groups. This indicated that both of the modifiers could promote the transformation of Pb, Zn, Cu, and Cd in tailings from the unstable acid-extractable and Fe-Mn binding state to the more stable organic binding state and residue state. The stabilization effect of mushroom residue on the four metals was better than that of calcium carbonate.

It can be seen from Figure 4 that the increase in modifier proportion can improve the stabilization degree of Pb, Zn, Cu, and Cd. The decreasing range of acid-extractable and Fe-Mn binding state content of Pb, Zn, Cu, and Cd and the increasing of organic binding and residue state content increased with the increase in modifier proportion. In the calcium carbonate treatment groups, the decreasing range of acid-extractable state content and the increasing range of residue state content of Pb, Zn, Cu, and Cd were Pb, Zn > Cu, Cd. The decreasing range of Fe-Mn binding state content and the increasing of organic binding state content were Pb, Cu > Zn, Cd. That indicated that the stabilization of calcium carbonate on Pb, Zn, Cu and Cd was Pb > Zn > Cu > Cd. In the mushroom residue treatment groups, the decreasing range of acid-extractable state content of Pb, Zn, Cu, and Cd was Pb > Zn > Cd > Cu. The decreasing range of Fe-Mn binding state content was Zn, Cd > Cu > Pb. The increasing range of organic binding state content was Cu > Pb > Cd > Zn, and the increasing range of residue state content was Zn > Cd > Pb > Cu. That indicated that the stabilization of mushroom residue on Pb, Zn, Cu, and Cd had the trend of Pb, Zn > Cd > Cu.

### 3.3. Effects of Different Modifiers and Their Gradients on Growth and Tolerance of Nerium indicum

#### 3.3.1. Effects of Different Modifiers and Their Gradients on *Nerium indicum* Biological Increment

Under different improvement gradients, the biomass increments of each organ and whole plant of *Nerium indicum* showed a trend of M3 > M2 > M1 > C3 > C2 > C1 > CK in Table 3. The biomass increment of mushroom residue improvement groups was 2.3~4.4 times as much as the control group (the whole plant biomass 108.82 ± 3.76 g/pot), while that of calcium carbonate improvement groups was 1.2~1.4 times as much as the control group. The results showed that the two modifiers could increase the biomass of *Nerium indicum*, and the effect of mushroom residue was better than calcium carbonate.

In the modified calcium carbonate groups, with the increase in the proportion of the modifier, there was no significant difference in biomass increment among the three treatment groups (*p* > 0.05). In the mushroom residue treatment groups, there was a significant difference when the additive amount was 30% (*p* < 0.05). This indicated that the biomass of *Nerium indicum* could not be significantly increased by increasing the proportion of calcium carbonate, while it could be significantly increased by increasing the proportion of the mushroom residue to 30%.

#### 3.3.2. Effects on Heavy Metal Content and Accumulation in Each Part of *Nerium indicum*

The contents of Pb and Zn in different organs of *Nerium indicum* treated with modifiers are shown in Table 4. Under different calcium carbonate or mushroom residue improvement gradients, Pb and Zn content in different organs of *Nerium indicum* showed a trend of root > stem > leaf. The addition of calcium carbonate significantly decreased the Pb and Zn content in the roots of *Nerium indicum* (Pb 38.49–65.44%, Zn 42.13–61.02%) (*p* < 0.01). However, the Pb content in the stems and leaves increased (stem 33.07–68.63%, leaf 42.77–78.22%) and the Zn content decreased (stem 4.84–42.99%, leaf 46.20–50.53%). In the mushroom residue treatment groups, the content of Pb and Zn in different organs of *Nerium indicum* was CK > improvement groups, and Pb content decreased significantly (root 35.32–42.41%, stem 34.66–77.34%, leaf 46.79–64.85%) (*p* < 0.01). The Pb content in roots, stems and leaves was 189.01, 43.66, and 8.87 mg/kg in M1 group, 197.28, 27.49, and 6.66 mg/kg in M2 group, and 212.27, 15.14, and 5.86 mg/kg in M3 group. The Zn content in roots, stems, and leaves were 322.58, 67.33, and 48.41 mg/kg in M1 group, 378.08, 55.87, and 41.70 mg/kg in M2 group, and 398.33, 49.79, and 38.04 mg/kg in M3 group. The concentration of Pb/Zn in root is inversely proportional to the proportion of modifier, while it is directly proportional in stems and leaves. The results showed that the addition of calcium carbonate could reduce the Zn content in all organs of *Nerium indicum*, reduce the Pb content in root, and increase the Pb content in stem and leaf. As shown in Table 4, the addition of mushroom residue could reduce the Pb/Zn content in all organs of *Nerium indicum*. From this, the addition of calcium carbonate and mushroom residue modifiers can reduce the pressure of Pb/Zn on *Nerium indicum*.

In all improvement gradients of the two kinds of improver, the accumulation of Pb and Zn in each organ of *Nerium indicum* showed the tendency of root > stem > leaf in Table 5. According to the control group, Pb and Zn in *Nerium indicum* were mainly concentrated in the root (Pb 71.85%, Zn 70.55%), and the accumulation of Zn (6.69 mg/pot) in *Nerium indicum* was significantly higher than that of Pb (4.44 mg/pot). In the calcium carbonate improved groups, the accumulation of Pb and Zn in the root of *Nerium indicum* revealed a phenomenon of a downward trend first and then an upward trend with the proportion (Pb: CK 3.19 mg/pot, C1 1.98 mg/pot, C2 2.81 mg/pot, C3 3.61, mg/pot and Zn: CK 4.72 mg/pot, C1 3.30 mg/pot, C2 4.16 mg/pot, C3 5.06 mg/pot). However, the accumulation of Pb in the stem and leaf is higher than the CK group, while the accumulation of Zn is lower than the CK group. It was consistent with the regularity of the content of Pb/Zn in each organ of *Nerium indicum*. The Pb/Zn content of all *Nerium indicum* organs in the mushroom residue improvement groups was higher than that in the control group (increase rate: 5.55–396.23%), which indicated that the addition of mushroom residue could improve the accumulation ability of Pb and Zn in *Nerium indicum*. Except Pb in stem, the accumulation ability is positively correlated with the proportion of mushroom residue.

#### 3.3.3. Effects on Bioconcentration Factor, Translocation Factor, and Transfer Factor of *Nerium indicum*

**Figure 5:**
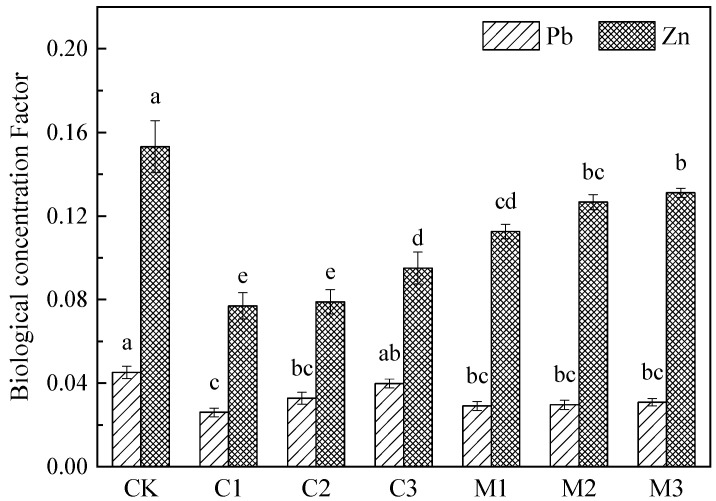
Biological concentration factor of *Nerium indicum* under improved treatment.

The biological concentration factor (BCF) of Pb and Zn in *Nerium indicum* under the two modifiers and their different improvement gradients are shown in Figure 5. The enrichment ability of *Nerium indicum* to Pb and Zn is weak, and the enrichment ability of Pb is weaker than Zn. The BCF of Pb and Zn in *Nerium indicum* showed a trend of CK > improvement groups (*p* < 0.01), indicating that the two modifiers can reduce the enrichment of Pb and Zn in *Nerium indicum*. Compared with the CK group, the BCF of Pb in C1 and M1 groups decreased by 42.29% and 35.49%, respectively. Compared with the CK group, the BCF of Zn in C1 and M1 groups decreased by 49.73% and 26.48%, respectively. However, the BCF of Pb and Zn increased with the modified proportion.

**Figure 6:**
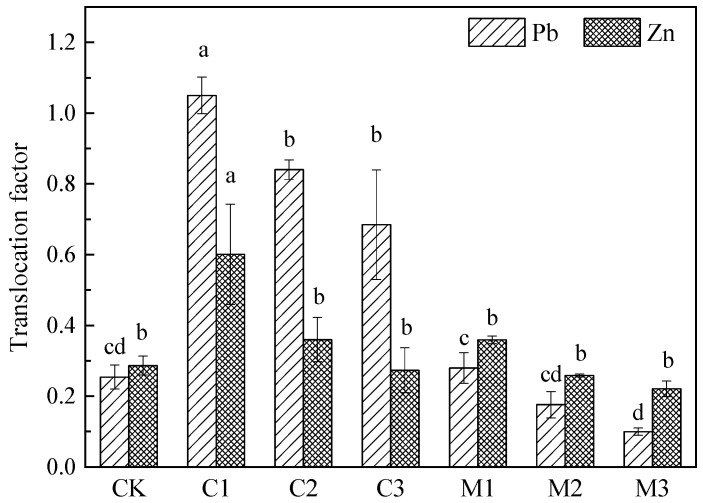
Translocation factor of *Nerium indicum* under calcium carbonate and mushroom residue improved treatment.

According to the translocation factor (TLF) of Pb and Zn in the *Nerium indicum* under two kinds of improvers and different improvement gradients in Figure 6, compared with the CK group, the BLF of Pb/Zn in C1, C2, and C3 groups increased by 313.51%, 230.77%, 169.49%, and 109.83%, 25.65%, −4.55%, respectively. The ability of *Nerium indicum* to transport Pb and Zn from underground to aboveground was enhanced after being treated with calcium carbonate, and the migration ability of Pb was stronger than that of Zn. Compared with the CK group, the BLF of Pb/Zn in M1, M2, and M3 groups increased by 10.04%, −30.81%, −60.89%, and 25.39%, −9.81%, −22.93%, respectively. On the contrary, the ability of *Nerium indicum* to transport Pb and Zn from underground to overground was weakened, and the migration ability of *Nerium indicum* to Zn was stronger than that of Pb in the mushroom residue improvement groups.

Compared with the control group, the TLF of *Nerium indicum* to Pb and Zn in the calcium carbonate improvement groups increased. The translocation factor of Pb was basically between 0.6 and 1.0, and that of Zn was basically between 0.2 and 0.6. This indicated that the ability of calcium carbonate to promote the migration of lead and zinc from underground to aboveground is enhanced. Compared with the control group, the TLF of Pb and Zn of *Nerium indicum* in the mushroom residue improvement groups was lower. The TLF of Zn was higher than that of Pb, but the TLF of Pb and Zn was still lower than 0.4. This indicated that the transport capacity of Pb and Zn in *Nerium indicum* improved by mushroom residue was very weak. However, the increase in two kinds of improver proportion can reduce the transportability of *Nerium indicum* to Pb and Zn. With the increase in modifier proportion, the TLF of Pb and Zn decreased, and there was no significant difference among the treatment groups.

**Figure 7:**
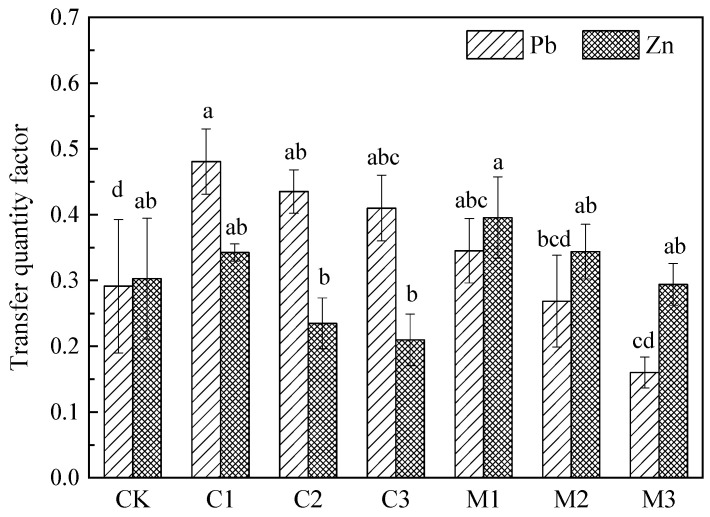
Transfer factor of *Nerium indicum* under calcium carbonate and mushroom residue improved treatment.

The transfer factor (TSF) of heavy metals is based on the heavy metal accumulation, which is most accurately defined as the percentage of the cumulative amount per unit time (year/season) per unit area (mu/ha) to the total absorption. It is usually used to measure the remediation capacity of plants for heavy metals in soil [37]. The transfer factor (TSF) of Pb and Zn in *Nerium indicum* is shown in Figure 7.

It can be seen that the Pb transfer factor of *Nerium indicum* showed a trend of calcium carbonate treatment groups (C1 0.48, C2 0.44, C3 0.41) > control group (CK 0.29). Pb content was low in all organs of *Nerium indicum*, but its aboveground biomass was significantly higher than the control group, which led to the accumulation of Pb in the aboveground part of *Nerium indicum* in the improved calcium carbonate groups higher than that in the control group. This increased the TSF of Pb in *Nerium indicum*. Except for the C1 group (C1 0.34), the Zn content in all organs of *Nerium indicum* in the C2 and C3 groups (C2 0.24, C3 0.21) was much lower than that in the control group (CK 0.30). Since zinc is mainly concentrated in the root, the accumulation of Zn in the aboveground part of *Nerium indicum* is lower than that in the control group. Pb/Zn transfer factor is inversely proportional to calcium carbonate concentration in the calcium carbonate treatment groups.

In the mushroom residue improvement groups, except for the 10% proportion treatment group (M1 0.35), the Pb transfer factor of *Nerium indicum* showed a trend of control group (CK 0.29) > mushroom residue treatment groups (M2 0.27, M3 0.16). The aboveground biomass of the improvement groups was significantly higher than that of the control group. Since Pb was mainly concentrated in the root, and the accumulation of Pb in the shoot was small, this led to the Pb accumulation of the control group being higher than the improvement groups. Except for the 10% proportion treatment group (M3 0.16), the Zn transfer factor of *Nerium indicum* showed a trend of mushroom residue treatment groups (M1 0.40, M2 0.34) > control group (CK 0.30). However, the biomass of the improvement groups was significantly higher than the control group, which led to the Zn accumulation in the aerial part of the improvement groups being higher than that of the control group. The Pb/Zn transfer factor is inversely proportional to mushroom residue concentration in the mushroom residue treatment groups.

In general, both calcium carbonate and mushroom residue can reduce the stress of Pb/Zn on *Nerium indicum*, promote the *Nerium indicum* biomass, and improve tolerance of *Nerium indicum* to Pb/Zn. These findings indicate that the phytoremediation technology of *Nerium indicum* combined with calcium carbonate or mushroom residue modifier has a good application potential in the ecological remediation of lead–zinc tailings [38].

## 4. Discussion

The abandoned land of lead–zinc tailings has a high content of heavy metals and low content of organic matter, which is not conducive to plant growth (Zhang et al. 2014). It is an effective way to restore the tailings field by planting tolerant plants combined with modifiers on it. In this study, the pH of the tailings increased after adding calcium carbonate. As a weak alkaline modifier, calcium carbonate can promote the hydrolysis of ${\mathrm{Al}}^{3+}$ in soil and neutralize $H^{+}$ produced by hydrolysis; thus, effectively increasing the soil pH. This is similar to the experimental research results of Guo [39]. The report of Chien showed that the higher the pH value of the soil within a certain range, the higher the adsorption capacity of the soil for heavy metals [40]. This may be because after calcium carbonate enters the soil, ${\mathrm{OH}}^{-}$ and ${\mathrm{CO}}_{3}^{2-}$ will form precipitation with heavy metal ions [41,42], which will stabilize the heavy metal ions in the soil; thus, reducing the contents of acid-extractable and Fe-Mn binding state of Pb and Zn with high activity, and increasing the contents of organic binding state and residue state with low activity. Soil enzyme activity is highly sensitive to heavy metals, which is an important index for evaluating soil health status and determining soil heavy metal content [43]. In this study, both of the modifiers can improve the activity of phosphatase and catalase in tailing, which is consistent with the increase in organic matter content and the decrease in unstable heavy metal content in tailing.

In this study, it was found that after calcium carbonate was applied, the content of Zn in organs of *Nerium indicum* substantially decreased, while the content of Pb in stems and leaves increased. This may be because the morphology of heavy metals in the soil directly affects the absorption of heavy metal ions by plants [44]; thus, affecting the transport of elements from plant roots to the aboveground, and the effects on different heavy metal elements vary to different degrees [45]. Moreover, the absorption of Pb and Zn in *Nerium indicum* was inhibited. This may be related to ${\mathrm{Ca}}^{2+}$ competing adsorption sites and ion channels with heavy metal ions after calcium carbonate is applied to the soil [46]. It may also be that the increase in soil pH after the application of calcium carbonate increases the negative charge on the surface of soil particles. That increases the adsorption strength of Pb and Zn, and forms a carbonate bound state and hydroxide precipitation; thus, reducing the migration of Pb and Zn ions in the soil and the absorption of heavy metals by crops [47]. The same results were also shown in the study of Zhao [48]. The decrease in Pb and Zn content in *Nerium indicum* reduced the stress degree of *Nerium indicum* under Pb and Zn stress and promoted the growth of *Nerium indicum*, so as its biomass increased [49]. At the same time, the accumulation of Pb and Zn in *Nerium indicum* increased. Therefore, the phenomenon of low concentration and high accumulation of Pb and Zn appeared in *Nerium indicum* [38].

As an improved material with high organic matter content, the mushroom residue can not only improve the physical and chemical properties of tailings, improve the water holding rate of the soil, chelate with heavy metal ions, but also reconstruct the microbial community while releasing nutrients [50,51,52]. The mushroom residue is rich in organic acids such as Fulvic acid and amino acid. These groups in the organic acids such as -COOH, -OH, and -CHO can react with heavy metal ions to form heavy metal–organic chelate [53] or generate precipitation, co-precipitation [54], etc. They may also make heavy metal ions directly adsorb on their surface [50] and reduce the content of forms with high activity of heavy metals, with active low residue in the soil. It was found that the addition of mushroom residue significantly increased the pH value of tailing, organic matter content, soil phosphatase, and catalase activities, and decreased the Pb and Zn contents in *Nerium indicum*, which was similar to the results of Zhang [21]. The study of Peng found that three kinds of organic industrial wastes decreased the heavy metal content in plants while increasing the nutrient element content in the substrate and increasing the soil enzyme activity, and the former and the latter showed a very significant negative correlation [55]. The effect of organic modifiers on vegetation restoration is obvious, which may be because the addition of organic modifiers can reduce the accumulation of heavy metals in plants and increase the biomass of plants after reducing the effective state content of heavy metals in the substrate. That reduces the concentration of heavy metals in plants and accumulation of heavy metals under the condition of ensuring the normal growth of plants [56,57].

## 5. Conclusions

Separate application of mushroom residue and calcium carbonate can effectively increase the pH value, organic matter content, and phosphatase and catalase activities of the tailing. It can also improve the stability of lead, zinc, copper, and cadmium in the tailing. The effect of mushroom tailings is better than that of calcium carbonate. Both the enzyme activity and metal stability in the tailings are positively correlated with the addition ratio of the modifier. Adding calcium carbonate can reduce the zinc content in all organs of *Nerium indicum*. The addition of mushroom residue can reduce the content of Pb and Zn in the organs of *Nerium indicum*, thereby reducing the stress of Pb and Zn on *Nerium indicum*, promoting the increase in *Nerium indicum* biomass, and improving the phytoremediation ability of *Nerium indicum*. In general, modifiers can effectively improve the adaptability and phytoremediation ability of *Nerium indicum* to heavy metal, and the effect of organic modifier mushroom residue is better than that of inorganic calcium carbonate.

## Figures and Tables

**Table 1:** Treatment of different substrates (weight ratio).

Treatment Group	The Ratio of the Substrate
Control group CK	100% tailing + 0.66 kg calcium magnesium phosphate fertilizer
Treatment group C1	95% tailing + 5% calcium carbonate + 0.66 kg calcium magnesium phosphate fertilizer
Treatment group C2	90% tailing + 10% calcium carbonate + 0.66 kg calcium magnesium phosphate fertilizer
Treatment group C3	85% tailing + 15% calcium carbonate + 0.66 kg calcium magnesium phosphate fertilizer
Treatment group M1	90% tailing + 10% mushroom residue + 0.66 kg calcium magnesium phosphate fertilizer
Treatment group M2	80% tailing + 20% mushroom residue + 0.66 kg calcium magnesium phosphate fertilizer
Treatment group M3	70% tailing + 30% mushroom residue + 0.66 kg calcium magnesium phosphate fertilizer

**Table 2:** Physical and chemical properties of soil used for pot test.

Substrates and Criteria	pH	Organic Matter (mg/kg)	Heavy Metal Content (mg/kg)			
			Pb	Zn	Cu	Cd
CK	7.21	25.95	4297.08	1704.92	163.50	43.83
C1	7.29	22.89	4135.58	1651.83	161.75	42.83
C2	7.31	22.69	3916.42	1573.25	153.00	40.00
C3	7.58	21.42	3894.17	1400.42	150.17	33.42
M1	7.24	31.71	4011.25	1613.83	158.17	39.50
M2	7.18	33.80	3702.25	1448.67	153.92	29.17
M3	6.95	37.66	3311.50	1398.33	143.33	28.92
National Ⅱ level standard	/	/	≤300	≤250	≤100	≤0.3
Chinese soil mean value	/	/	26.0	74.2	22.6	0.097
Soil background value of Hunan province	/	/	26.3	90.0	25.0	0.081

**Table 3:** *Nerium indicum* biomass increase in different organs with different modified treatments.

Improved Gradient	Root Biomass (g/Pot)	Stem Biomass (g/Pot)	Leaf Biomass (g/Pot)	The Whole Plant Biomass (g/Pot)
CK	30.06 ± 5.20 c	47.89 ± 5.99 d	30.86 ± 1.71 c	108.82 ± 3.76 c
C1	48.89 ± 7.44 bc	49.56 ± 4.14 d	33.36 ± 2.20 c	131.83 ± 13.68 c
C2	54.09 ± 14.40 b	55.06 ± 20.09 cd	33.86 ± 2.86 c	143.02 ± 34.12 c
C3	53.29 ± 6.33 b	56.73 ± 10.53 cd	42.20 ± 6.65 c	152.22 ± 23.32 c
M1	56.30 ± 4.27 b	96.33 ± 24.25 bc	100.56 ± 31.54 b	253.20 ± 58.85 b
M2	58.40 ± 10.13 b	115.70 ± 18.70 b	126.03 ± 12.47 b	300.13 ± 36.26 b
M3	98.33 ± 10.60 a	177.66 ± 24.76 a	200.80 ± 23.75 a	476.80 ± 48.11 a

**Table 4:** Heavy metal content in different organs of *Nerium indicum*.

Treatment Group	Heavy Metal Content in Each Organ (mg/kg)					
		Pb			Zn	
	Root	Stem	Leaf	Root	Stem	Leaf
CK	328.21 ± 6.35 a	66.82 ± 12.15 bc	16.67 ± 0.54 c	487.08 ± 32.07 a	88.58 ± 19.64 a	51.12 ± 2.21 a
C1	113.44 ± 14.69 d	88.92 ± 6.438 ab	29.71 ± 3.91 a	189.83 ± 28.93 f	84.29 ± 15.48 ab	27.5 ± 7.43 cd
C2	154.37 ± 11.82 c	104.20 ± 15.21 a	25.68 ± 2.62 ab	224.83 ± 22.91 ef	53.75 ± 12.58 bc	26.33 ± 5.50 cd
C3	201.87 ± 17.54 b	112.68 ± 19.35 a	23.80 ± 1.15 b	281.83 ± 38.86 de	50.5 ± 18.01 bc	25.29 ± 5.05 d
M1	189.01 ± 17.82 bc	43.66 ± 7.11 cd	8.87 ± 0.42 d	322.58 ± 10.66 cd	67.33 ± 3.88 abc	48.41 ± 2.02 ab
M2	197.28 ± 21.38 b	27.49 ± 5.14 de	6.66 ± 0.99 d	378.08 ± 26.12 bc	55.87 ± 4.18 abc	41.70 ± 1.58 ab
M3	212.27 ± 18.20 b	15.14 ± 2.52 e	5.86 ± 0.92 d	398.33 ± 5.29 b	49.79 ± 2.75 c	38.04 ± 6.07 bc

**Table 5:** Heavy metal accumulation in different organs of *Nerium indicum*.

Treatment Group	Heavy Metal Accumulation in Each Organ (mg/Pot)					
	Pb			Zn		
	Root	Stem	Leaf	Root	Stem	Leaf
CK	3.19 ± 0.84 ab	1.08 ± 0.34 a	0.17 ± 0.00 b	4.72 ± 1.25 c	1.44 ± 0.51 a	0.53 ± 0.06 c
C1	1.98 ± 0.43 c	1.49 ± 0.07 a	0.32 ± 0.03 ab	3.30 ± 0.57 c	1.41 ± 0.25 a	0.30 ± 0.09 c
C2	2.81 ± 0.80 bc	1.90 ± 0.82 a	0.29 ± 0.04 ab	4.16 ± 1.56 c	0.91 ± 0.10 a	0.30 ± 0.08 c
C3	3.61 ± 0.71 bc	2.13 ± 0.06 a	0.33 ± 0.04 ab	5.06 ± 1.25 c	0.94 ± 0.26 a	0.36 ± 0.13 c
M1	3.45 ± 0.83 bc	1.50 ± 0.10 a	0.26 ± 0.07 ab	5.86 ± 1.11 c	2.34 ± 0.18 b	1.45 ± 0.42 b
M2	4.41 ± 0.38 b	1.34 ± 0.45 a	0.28 ± 0.07 ab	8.46 ± 0.43 b	2.66 ± 0.52 b	1.82 ± 0.50 ab
M3	8.07 ± 0.94 a	1.14 ± 0.35 a	0.40 ± 0.07 a	15.12 ± 0.45 a	3.70 ± 0.45 a	2.63 ± 0.64 a

## Data Availability

The authors confirm that the data supporting the findings of this study are available within the article.
